# Lagging X chromatids specify the orientation of asymmetric organelle partitioning in XX spermatocytes of *Auanema rhodensis*

**DOI:** 10.1093/genetics/iyac159

**Published:** 2022-10-18

**Authors:** Talal Al-Yazeedi, Emily L Xu, Jasmin Kaur, Diane C Shakes, Andre Pires-daSilva

**Affiliations:** School of Life Sciences, University of Warwick, Coventry CV4 7AL, UK; Department of Biology, William & Mary, Williamsburg, VA 23187, USA; School of Life Sciences, University of Warwick, Coventry CV4 7AL, UK; Department of Biology, William & Mary, Williamsburg, VA 23187, USA; School of Life Sciences, University of Warwick, Coventry CV4 7AL, UK

**Keywords:** asymmetric cell division, meiosis, *Caenorhabditis elegans*, *Auanema*, MSP, mitochondria, spermatogenesis, nematode, X chromosome

## Abstract

The unequal partitioning of molecules and organelles during cell division results in daughter cells with different fates. An extreme example is female meiosis, in which consecutive asymmetric cell divisions give rise to 1 large oocyte and 2 small polar bodies with DNA and minimal cytoplasm. Here, we test the hypothesis that during an asymmetric cell division during spermatogenesis of the nematode *Auanema rhodensis*, the late segregating X chromatids orient the asymmetric partitioning of cytoplasmic components. In previous studies, the secondary spermatocytes of wild-type XO males were found to divide asymmetrically to generate functional spermatids that inherit components necessary for sperm viability and DNA-containing residual bodies that inherit components to be discarded. Here we extend that analysis to 2 novel contexts. First, the isolation and analysis of a strain of mutant XX pseudomales revealed that such animals have highly variable patterns of X-chromatid segregation. The pattern of late segregating X chromatids nevertheless predicted the orientation of organelle partitioning. Second, while wild-type XX hermaphrodites were known to produce both 1X and 2X sperm, here, we show that spermatocytes within specific spermatogonial clusters exhibit 2 different patterns of X-chromatid segregation that correlate with distinct patterns of organelle partitioning. Together this analysis suggests that *A. rhodensis* has coopted lagging X chromosomes during anaphase II as a mechanism for determining the orientation of organelle partitioning.

## Teaser

In males of the nematode *Auanema rhodensis*, only X-bearing sperm are viable because the secondary meiotic division is asymmetric: the X chromosome co-segregates with organelles necessary for sperm function. The other cell (nullo-X) becomes a residual body. Here, Al-Yazeedi, Xu, *et al*. show that XX pseudomales and XX hermaphrodites have variable segregation patterns of the X chromosome. Since the organelles necessary for sperm function consistently co-segregate with the X chromosome, this chromosome likely acts as an asymmetric signal.

## Introduction

The laws of genetics presume that meiotic chromosome segregation is both highly accurate and unbiased. Yet, nonrandom and/or unequal chromosome segregation has been well-documented in multiple species (Pardo-Manuel de Villena and Sapienza 2001; Pajpach *et al.* 2021; Van Goor, Shakes, *et al.* 2021). Such deviations from Mendelian expectations are typically associated with oocyte meiosis. During oocyte meiosis, highly asymmetric cell divisions yield a single functional gamete, while the other meiotic products are discarded as diminutive polar bodies (Dalton and Carroll 2013). This asymmetry opens a potential for biased segregation if certain chromosomes can preferentially segregate to the functional oocyte (Pardo-Manuel de Villena and Sapienza 2001; Kruger and Mueller 2021). In instances when biased chromosome segregation involves a sex chromosome, biased segregation can result in subtle or dramatically skewed sex ratios (Jaenike 2001).

**Fig. 1.**
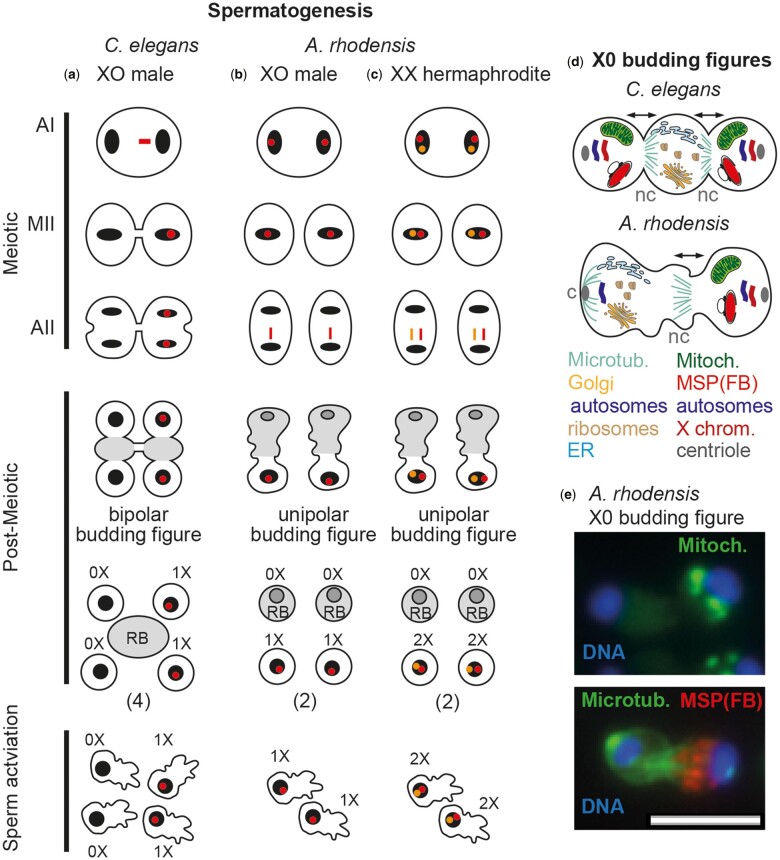
Comparison of spermatogenesis between *C. elegans* and *A. rhodensis*. a) In *C. elegans* males, the unpaired X-chromosome (in red) lags during anaphase I, leading to the formation of 2 secondary spermatocytes that divide symmetrically to generate 2 gametes with only autosomes (0X) and 2 gametes with autosomes plus a single X (1X). A residual body (RB), indicated in gray, is formed in the postmeiotic phase that follows anaphase II. b) In *A. rhodensis* males, the sister chromatids of the X chromosome separate and segregate during meiosis I such that each secondary spermatocyte receives 1 X chromatid. During anaphase II, the unpaired X-chromatid lags before joining the autosomes of the functional sperm. In a modified, unipolar partitioning process, the non-X-chromosome set ends up in the residual body. c) During spermatogenesis in *A. rhodensis* hermaphrodites, the homologous Xs (red, orange) fail to pair, and the subsequent segregation patterns result in the formation of sperm with 2 nonsister X chromatids (2X). d) Schematic comparing the bipolar and unipolar partitioning events that follow anaphase II during *C. elegans* and *A. rhodensis* male spermatogenesis, respectively. Arrows indicate that partitioning in *C. elegans* is bipolar and unipolar in *A. rhodensis*. Materials required for sperm motility like mitochondria and the sperm cytoskeleton protein MSP partition to the functional sperm whereas ribosomes, ER, and the Golgi complex partition to the residual body. In *C. elegans* spermatocytes, α-tubulin complexes separate bilaterally from the anaphase II centrosome to establish 2 noncentrosomal (nc) microtubule arrays, 1 at each spermatid-residual body junction. In *A. rhodensis*, this conversion occurs only on the side of the functional sperm, resulting in 1 nc mitrotubule array while microtubules at the other pole remain centrosomal (c) (Winter *et al.* 2017). e) Immunofluorescence images of indicated components in *A. rhodensis* partitioning stage spermatocytes. Scale bar = 5 µm.

Spermatocyte meiosis is not usually associated with this type of biased chromosome segregation since the meiotic divisions of individual spermatocytes typically yield 4 equal-sized gametes. As the heterogametic sex, males produce equal numbers of 2 distinct types of gametes: X- and Y-bearing sperm in the case of humans or 1X and 0X-bearing sperm in the case of most nematodes. For example, in XO *Caenorhabditis elegans* males, the unpaired X-chromosome lags during meiosis I and then segregates to one of the 2 secondary spermatocytes (Albertson and Thomson 1993; Shakes *et al.* 2009) (Fig 1a). During meiosis II, the X-bearing secondary spermatocyte divides to form 2 X-bearing sperm, whereas the non-X-bearing spermatocyte divides to form 2 non-X-bearing sperm.

All sperm differentiation programs include a postmeiotic, asymmetric partitioning step during which sperm are streamlined by discarding components that are no longer needed into a residual body (RB) (Breucker *et al.* 1985; Steinhauer 2015). Furthermore, the underlying cell polarization and scission events in humans (de Kretser *et al.* 1998), *Drosophila* (Noguchi *et al.* 2006; Steinhauer *et al.* 2019), and *C. elegans* (Ward *et al.* 1981; Kelleher *et al.* 2000; Winter *et al.* 2017; Hu *et al.* 2019) all involve common elements such as actin, myosin VI, and microtubules (Zakrzewski *et al.* 2021). Specific to nematode spermatogenesis, RB formation occurs surprisingly early, immediately after anaphase II (Fig. 1a) (Chu and Shakes 2013). Presumably because of this juxtaposition, the partitioning machinery coopts the anaphase II axis to establish an RB between the 2 spermatids (Fig. 1, a and d) (Winter *et al.* 2017).

Spermatocyte meiosis in males of the nematode *Auanema rhodensis* (aka, *Rhabditis* sp. SB347) (Kanzaki *et al.* 2017) deviates from this pattern in 3 key ways: (1) during meiosis I, the unpaired X chromosome splits into sister chromatids, (2) during meiosis II, the individual X chromatids segregate to the pole that will become functional sperm, and (3) in the postmeiotic phase, what is typically a bipolar partitioning process becomes unipolar process (Shakes *et al.* 2011; Winter *et al.* 2017; Tandonnet *et al.* 2018) (Fig. 1, b, d, and e)*.* Thus, meiotically dividing *A. rhodensis* spermatocytes yield 2 functional 1X sperm and 2 residual bodies containing the non-X-bearing DNA complement (Shakes *et al.* 2011; Winter *et al.* 2017). These deviations lead to highly skewed sex ratios in *A. rhodensis* (Félix 2004; Shakes *et al.* 2011; Tandonnet *et al.* 2018), as crosses between XO males and XX females produce mostly XX offspring from the union of 1X oocytes with 1X sperm.

In *A. rhodensis*, deviations from Mendelian X-chromosome patterns extend beyond male spermatogenesis. XX females follow Mendelian predictions and produce mostly 1X oocytes; however, XX hermaphrodites produce 0X oocytes and mostly 2X sperm (Fig. 1c) (Tandonnet *et al.* 2018). Importantly, these unusual X-chromosome segregation patterns during hermaphrodite oogenesis and spermatogenesis correlate with an absence of X-chromosome recombination.

Variant modes of X-chromosome segregation may be necessary for XO males to produce exclusively 1X sperm and XX hermaphrodites to produce mostly 2X sperm, but they are not sufficient (Edwards 1910; Winter *et al.* 2017). Also, critical is the conversion of a bipolar partitioning process into a unipolar process such that half of the genetic material is discarded in residual bodies. We suspect that the diminutive size of *A. rhodensis* spermatocytes may necessitate halving the number of functional sperm that can be produced with sufficient mitochondria and motility proteins (Winter *et al.* 2017). Importantly, the almost exclusive production of X-bearing sperm also requires that materials required for sperm function are consistently partitioned to the side with X-bearing chromatin mass.

Based on our previous work, we hypothesized that the late segregating X chromatid(s) in *A. rhodensis* acts during the second meiotic division to dictate the direction of the asymmetric partitioning process. In this study, we critically tested this hypothesis by examining patterns of X-chromosome behavior and organelle partitioning under 2 exceptional circumstances. First, we isolated a sex determination mutant in which XX animals are phenotypically transformed into fertile males to examine how X chromosomes and organelle partitioning might occur in the abnormal cellular background of an XX male germline. Second, we examined the natural variation in the spermatogenesis program of wild-type self-fertilizing *A. rhodensis* hermaphrodites that enable them to produce a small number of male offspring, particularly early in the brood (Chaudhuri *et al.* 2015). These male offspring are inferred to be the products of nullo-X oocytes and 1X sperm (Tandonnet *et al.* 2018), and we hypothesize that the capacity of wild-type *A. rhodensis* hermaphrodites to produce small numbers of 1X sperm is related to the fact that they produce sperm in discrete spermatogonial clusters throughout adulthood (McCaig *et al.* 2017). Here, we reveal the variable patterns of X-chromosome segregation in the spermatocytes of both XX pseudomales and wild-type XX hermaphrodites and show that in both cases, the pattern of lagging X chromatids predicts the pattern of organelle partitioning.

## Materials and methods

For materials, see Table 1.

**Table 1.** Reagent table.

Data type	Experimental species	Symbol/name used in publication	Source—public	Source—published	Source—unpublished	Identifiers	New reagent	Comments
Data type (mandatory) duplicate rows as needed. Order is flexible, but row titles must be preserved	Experimental species (mandatory, “NA” okay)	Symbol/name used in publication (mandatory)	Source—public [stock center; company, data repository] (one of D, E, F mandatory)	Source—published (PMID or “this paper”) (one of D, E, F mandatory)	Source—unpublished (description, incl. lab of origin) (one of D, E, F mandatory)	Identifiers (format as ID_source: identifier) Separate multiple entries with semicolon, space	New reagent (mandatory for new entities) Description, progenitor(s)	Comments (optional) Genotypes, purpose of reagent, additional information
genetic reagent (in whole organism)	*Auanema rhodensis*	*A. rhodensis*, APS4 strain	Warwick University, Pires lab.	Kanzaki *et al.* (2017)				Mutagenesis was conducted on the APS4 strain to obtain *Arh-mas-1* mutant. *Arh-mas-1* psedumale was crossed with APS4 female to quantify sex of offspring. Gonad of *Arh-mas-1* identified in the APS4 strain was used for cytological studies.
genetic reagent (in whole organism)	*Auanema rhodensis*	*A. rhodensis*, APS6 strain	Warwick University, Pires lab.	Kanzaki *et al.* (2017)				APS6 strain was used as a perantal line for backcrossing experiments with *Arh-mas-1 *line. *Arh-mas-1* psedumales was crossed with APS6 females and genotyping of the progeny elucidate the gametes produced by *Arh-mas-1* psedumales.
antibody	NA	FITC-conjugated anti-α-tubulin (mouse)	Sigma Aldrich (DM1A)					Immunocytology of *Arh-mas-1* sperm spread
antibody	NA	G3197 anti-MSP monoclonal (rabbit)		Kosinski *et al.* (2005)				Immunocytology of *Arh-mas-1* sperm spread
antibody	NA	Anti-CYP33E1	Developmental Studies Hybridoma Bank	Hadwiger *et al.* (2010)				Immunocytology of *Arh-mas-1* sperm spread
antibody	NA	Anti-smo-1 (sumo) (mouse)	Developmental Studies Hybridoma Bank	Pelisch *et al.* (2014)				Immunocytology of Arh-*mas-1* sperm spread
antibody	NA	3D5 anti-ATPB (mouse)	Abcam					Immunocytology of *Arh-mas-1* sperm spread
antibody	NA	Alexa Fluor Plus 555-conjugated goat antirabbit IgG (goat)	Invitrogen					Immunocytology of *Arh-mas-1* sperm spread
antibody	NA	Alexa Fluor 488 goat-antimouse IgG (H + L) (goat)	Jackson ImmunoResearch Laboratories			AB_2338840		
commercial assay	NA							
chemical compound, drug	NA	DAPI	Electron Microscopy Sciences					Immunocytology of *Arh-mas-1* sperm spread
Chemical compound, drug	NA	Ethyl methane sulfonate	Sigma Aldrich	Pires-daSilva and Sommer (2004)		M0880		The chemical was used to mutagenize APS4, *A. rhodensis* strain to identify *Arh-mas-1* mutant

### Nematodes strains and cultures

The *A. rhodensis* inbred strains APS4 and APS6 (Tandonnet *et al.* 2018) were maintained according to standard conditions for *C. elegans*, at 20°C (Stiernagle 2006). Nematodes were cultured on NGM plates seeded with either the *Escherichia coli* strain OP50 or the streptomycin-resistant strain OP50-1.

### Mutagenesis

*A. rhodensis* APS4 was mutagenized with the chemical mutagen ethyl methanesulfonate, as previously described (Pires-daSilva and Sommer 2004; Chaudhuri *et al.* 2011). To screen for a masculinizing phenotype (XX pseudomales), 521 F1 (dauer) hermaphrodites from mutagenized P0s were individually transferred to single plates and allowed to self-fertilize. To simplify screening for mutants that generate high rates of (pseudo)males, we transferred the individual F1s to new plates as 3-day-old adults, to identify individuals who produced large numbers of F2 males. We adopted this procedure because *A. rhodensis* hermaphrodites of this age produce fewer XO self-offspring (∼3%) than younger hermaphrodites (∼8%) (Chaudhuri *et al.* 2015). From late brood plates scored as having potential pseudomales, 10–15 sibling hermaphrodites were isolated to single plates to maintain the mutation as a heterozygous strain (Supplementary Fig. 1). Heterozygous hermaphrodites were selected based on the production of excess male offspring, which is consistent with the anticipated production of 25% male offspring. The mutant strain was backcrossed with the wild-type APS4 strain for 3 generations to remove background mutations generated during the mutagenesis. The *A. rhodensis* masculinizer was named *Arh-mas-1* (*brz-3*), following the nomenclature described in Wormbase (www.wormbase.org).

### Single nematode genotyping

Single-nucleotide polymorphisms that distinguish the X chromosomes of the APS4 and APS6 strains (markers 9686 and 12469) were used to determine the origin of the X in XO males and verify that pseudomales were XX animals. These genetic markers together with the primer sequences, restriction enzymes and fragment sizes are detailed in (Tandonnet *et al.* 2019) and at https://data.mendeley.com/datasets/63d7rrrx28/3#file-16ff094d-6c74-478a-a3f5-8878e89fd72f.

### Crosses and brood counts

To isolate virgin APS6 females for crosses, L4 larvae from the first 12–24 h of a hermaphrodite brood were picked to individual plates. Females were distinguished from their hermaphrodite siblings due to their faster sexual maturation and lack of self-fertility (Kanzaki *et al.* 2017).

To determine whether a potential *mas-1* pseudomale had 2 X chromosomes, individual APS4-derived pseudomales were crossed to individual APS6 females for 24 h at 20°C. Heterozygous (*mas-*1/+; X^APS4^/X^APS6^ F1) hermaphrodites from this cross were allowed to self-fertilize. The resulting F2 male offspring (either XO males or *mas-1*/*mas-1* pseudomales) were analyzed by single worm PCR to determine the genotype of their X chromosome(s).

To quantify the broods of *mas-1* males, individual putative APS4 *mas-1* males were allowed to mate with individual APS6 verified females for 24 h. After 24 h, the *mas-1* male was removed from the plate and dissected to confirm that the males were *mas-1*/*mas-1* pseudomales through the cytological analysis of its gonad (see *Immunocytology*). Individual mated APS6 females were daily transferred to new plates to assess the nature of their offspring (male, feminine, or Dpy).

### Immunocytology

Specimen preparation and antibody labeling followed established protocols (Shakes *et al.* 2009). Individual gonads were obtained by dissection of individual males (or hermaphrodites) in 5–10 μl of Edgar’s buffer (Edgar 1995). In most cases, sperm spreads to analyze detached spermatocytes were cracked in liquid nitrogen and were fixed overnight in −20°C methanol. However, anti-CYP33E1 samples were fixed in room temperature methanol for 60–90 min, and anti-ATPB samples were fixed in 4% paraformaldehyde and posttreated with Triton-X 100. Primary antibodies included: 1:100 FITC-conjugated anti-α-tubulin (DM1A—Sigma); 1:500 G3197 rabbit anti-major sperm protein (MSP) monoclonal (Kosinski *et al.* 2005); 1:100 anti-CYP33E1 (Developmental Studies Hybridoma Bank; Hadwiger *et al.* 2010); 1:100 mouse anti-SMO-1 (sumo) (Developmental Studies Hybridoma Bank 6F2; Pelisch *et al.* 2014); and 1:100 3D5 mouse anti-ATPB (Abcam). All samples were incubated with primary antibodies for 60–90 min at room temperature. Affinity-purified secondary antibodies included 1:400 Alexa Fluor Plus 555-conjugated goat antirabbit IgG (Invitrogen) and 1:100 Alexa Fluor 488 goat-antimouse IgG (H + L) (Jackson ImmunoResearch Laboratories).

Final slides were mounted with DAPI containing Fluoro Gel II mounting medium (Electron Microscopy Sciences). Single-focal plane images were acquired under epifluorescence using an Olympus BX60 microscope equipped with a QImaging EXi Aqua CCD camera. Photos were taken, merged, and exported for analysis using the program iVision. The levels adjust function in Adobe Photoshop was used to spread the data containing regions of the image across the full range of tonalities.

For the quantification of DNA intensity, sperm spreads were colabeled with DAPI and anti-MSP antibodies. Spermatids were chosen for quantification based on their DNA morphology and the presence of MSP. NIH ImageJ was used to determine integrated intensity (Xu 2020). For comparisons between DNA and FB segregation in partitioning stage *mas-1* spermatocytes, asymmetries were calculated as the integrated intensity of 1 chromatin mass (or FB region)/sum of the 2 integrated intensities. The Pearson correlation coefficient (*r*) was determined by Excel.

## Results

### *Arh-mas-1* has a male phenotype and XX karyotype

**Fig. 2.**
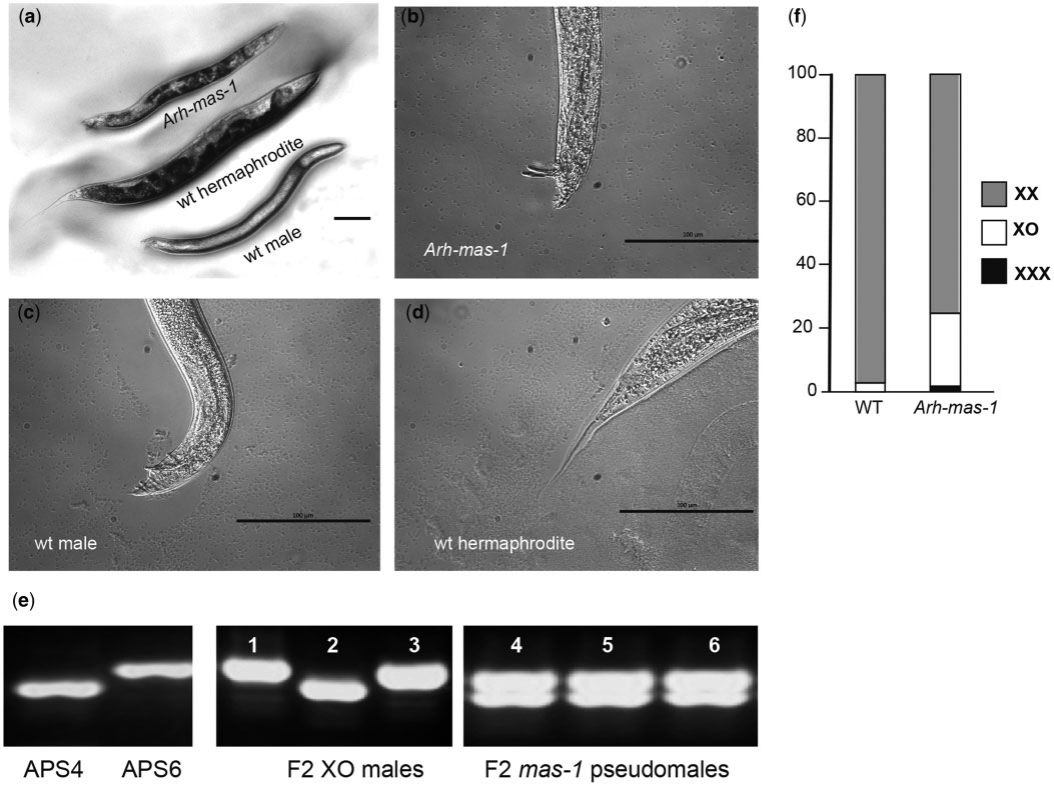
Arh-mas-1 masculinizes XX animals. a) The *Arh-mas-1* pseudomale has a dark gut pigmentation pattern. Bar, 100 µm. The tails of the *Arh-mas-1* (b) and male (c) are blunt and with spicules, whereas from the hermaphrodite (d) is long and slender. Bar, 100 µm. e) Genotyping of the X chromosome (chromosome marker 9686) in F2 (1–3) and F2 (4–6) animals from crosses between pseudomales (APS4 background) and females (APS6). f) Bar graph representing the proportion of XX animals (females or hermaphrodites), XO (males), and XXX (dumpy). Nineteen crosses were performed between APS4 wild-type males and females (*N* = 5,629 offspring), and 23 crosses were performed between *Arh-mas-1* pseudomales and APS4 females (*N* = 3,135 offspring).

To isolate a masculinizing mutant that would enable us to analyze X-chromosome segregation and organelle partitioning in the unique and unusual context of an XX male, we performed chemical mutagenesis and a genetic screen. From ethyl methanesulfonate mutagenized P0s, we screened for heterozygous F1 hermaphrodites that produced significant numbers (∼25%) of male offspring in the late portion of their broods, rather than the smaller number of males routinely produced by wild-type hermaphrodites in the initial portion of their broods (Chaudhuri *et al.* 2015) (Supplementary Fig. 1). From this screen, we isolated the sex determination mutant (*Arh-mas-1*) in which homozygous XX animals exhibit a male phenotype that is almost indistinguishable from XO wild-type males (Fig. 2a). Unlike similar mutants in other nematode species (Hodgkin and Brenner 1977; Pires-daSilva and Sommer 2004; Kelleher *et al.* 2008), *Arh-mas-1* pseudomales do not show signs of partial feminization. Instead, *Arh-mas-1* pseudomales have normal male reproductive structures, including a single-arm gonad and morphologically normal tail (Fig. 2, b–d). They also exhibit normal male mating behavior and are fertile when crossed with wild-type females. The molecular characterization of this mutant will be published somewhere else.

In the absence of genetic balancers, we propagated the recessive mutation through heterozygous hermaphrodites (Supplementary Fig. 1). In addition, although young adult *Arh-mas-1* XX pseudomales are indistinguishable from their XO wild-type (or heterozygous) counterparts, older *mas-1* pseudomales display a distinct gut pigmentation pattern, which we used as a marker to distinguish the 2 karyotypes (Fig. 2a). We do not know if this gut phenotype is related to the *mas-1* mutation or represents a second tightly linked genetic mutation that was not eliminated through backcrossing.

To confirm the XX karyotype of pseudomales, we crossed *Arh-mas-1* pseudomales (derived from the APS4 strain) to wild-type females of the independently isolated APS6 strain. Using markers for a single-nucleotide polymorphism on the X chromosome, we then genotyped F2 self-offspring from F1 hybrid (*mas-1*/+; X^APS4^/X^APS6^) hermaphrodites (Fig. 2e, samples 1–6). Numerous *Arh-mas-1* pseudomales were found to be heterozygous for the X-chromosome markers (Fig. 2e, samples 4–6), confirming that these pseudomales have an XX karyotype and that *mas-1* maps to an autosome.

### *mas-1* males sire offspring using mostly 1X and some 0X sperm

In genetic crosses with wild-type females, the composition of offspring sired by wild-type XO males and *mas-1* XX males differed (Fig. 2f). In both cases, most of the offspring were XX, reflecting the fertilization of 1X oocytes by 1X sperm. However, *mas-1* males sired many more male offspring. As we previously showed (Tandonnet *et al.* 2018), wild-type males produce exclusively 1X sperm, and the small number of XO male offspring produced in male–female crosses originate from rare 0X oocytes produced in female meiotic divisions. However, only 13/57 (23%) of XO male offspring sired by *mas-1* males inherited the paternal X (Fig. 2e, sample 6), while the remaining 44/57 (77%) inherited the maternal X (Fig. 2e, samples 4 and 5). These results indicate that *mas-1* XX pseudomales produce sizable numbers of functional 0X sperm. *mas-1* pseudomales also sired small numbers of Dpy offspring, presumably the product of 2X sperm and analogous to the Dpy XXX animals with dosage compensation defects that have been well documented in *C. elegans* (Hodgkin 1979; Vargas *et al.* 2017). Notably, in multiple independent brood studies of *mas-1* males, dead embryos indicative of autosomal aneuploidy were never observed; instead, chromosome variation appears to be restricted to the X chromosome.

### Cytological studies suggest *Arh-mas-1* XX pseudomales produce 0–4X sperm

**Fig. 3.**
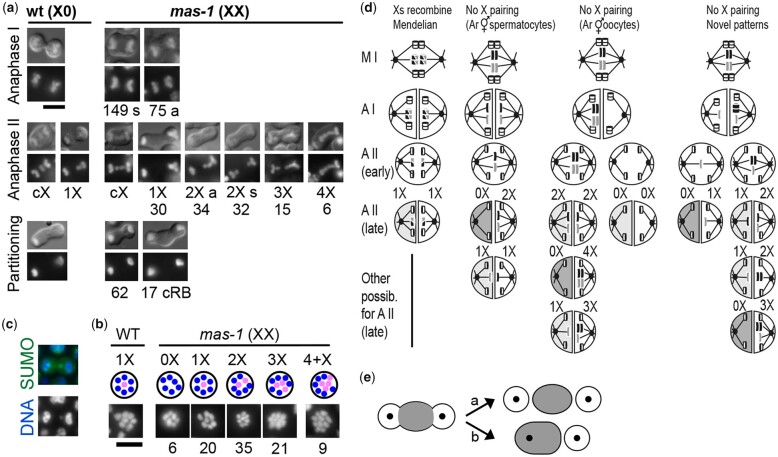
Variable X-chromosome segregation patterns in the spermatocytes of *mas-1* XX pseudomales. a) Comparison of anaphase X-chromosome segregation in wild-type XO males and *mas-1* XX pseudomales. Spermatocytes are visualized by differential interference contrast optics and Hoechst-stained chromosomes; dual images (top) and Hoechst only (bottom). Images are organized by stage from top to bottom. For individual images, the number of spermatocytes scored as having that pattern is indicated. Lagging X chromosomes/chromatids are described as centered (c) or evaluated as to their number (1–4X). If X chromosomes/chromatid segregate equally to both daughter cells, the division is symmetric (s); unequal segregation patterns are asymmetric (a). Cells oriented with likely RB on the left. Scale bar= 5 µm. b) Rare anaphase I spermatocyte with lagging chromosome colabeled with DAPI and anti-sumo antibody. c) A variable number of chromosomes in metaphase I plates, stained with DAPI. *N* = number of spermatocytes observed for each category, out of a total of 91. In schematics, X chromosomes/chromatids are in pink. Scale bar= 5 µm. d) Models for possible meiotic events in XX *mas-1* pseudomales. Homologous X chromosomes are depicted in grey and black, and autosomes are shown in white. X-chromosome recombination is indicated by mixed shaded chromosomes. In the models, the anaphase II chromosome set with fewer chromosomes is discarded in a residual body (dark gray cytoplasm). Even when X chromatids segregate equally or with a partial asymmetry, one of the 2 sets may end up in a residual body (light gray cytoplasm). e) Schematic of how budding figures with a cRB could potentially yield either 1 or 2 functional sperm.

The finding that XX *mas-1* males sire offspring mostly using 1X and 0X sperm was surprising, as wild-type XX *A. rhodensis* hermaphrodites produce mostly 2X sperm (Tandonnet *et al.* 2018) (Fig. 1c). To understand how X chromosomes were segregating within the distinct context of XX *mas-1* spermatogenesis, we examined the cytology of the meiotically dividing spermatocytes. Although the tools for directly marking the X chromosome are not available for *A. rhodensis*, meiotic spermatocytes imaged using differential interference contrast (DIC) and Hoechst revealed highly variable patterns of lagging X chromosomes in *mas-1* XX spermatocytes (Fig. 3a). As previously reported and in contrast to *C. elegans* males (Fig. 1a), we never observed lagging chromosomes during anaphase I in the spermatocytes of wild-type *A. rhodensis* males. Instead, the X-chromatid lags during meiosis II and remains in the center (c) before eventually segregating into one of the 2 autosome sets (Figs. 1b and 3a) (Shakes *et al.* 2011). In the spermatocytes of XX *Arh*-*mas-1* pseudomales, we rarely observed centrally positioned, lagging chromosomes during anaphase I. However, examination of DAPI intensity revealed that while 66% (*N* = 224) of the primary spermatocytes segregated DNA symmetrically, 33% (*N* = 224) segregated unequal amounts of DNA to their daughter cells. To increase the chance of documenting a rare lagging chromosome, we imaged fixed cells labeled with an anti-sumo antibody which labels chromosomes in *C. elegans* (Pelisch *et al.* 2014) and identified rare examples of lagging chromosome during anaphase I (Fig. 3b).

During anaphase II, secondary spermatocytes of *mas-1* pseudomales routinely exhibited a variety of lagging chromosome patterns with 1–4X chromatids segregating either symmetrically (s) or asymmetrically (a) to the 2 autosomal sets (Fig. 3a). Symmetric segregation patterns were most common when anaphase II spermatocytes had 2 X chromatids. This analysis also revealed a subset (∼20%, *N* = 79) of *mas-1* spermatocytes during the partitioning stage that formed “classic” bipolar budding figures with 2 budding spermatids flanking a central residual body (cRB). Consistent with the hypothesis that lagging X chromatids specify the orientation of the partitioning process, we found that this bipolar pattern and formation of cRBs was associated with symmetrically partitioning X chromosomes. We suspected that these bipolar figures also form when secondary spermatocytes lacked X chromatids altogether. While this morphology is the norm for most nematode spermatocytes including those of *C. elegans* (Fig. 1a), partitioning stage spermatocytes of wild-type *A. rhodensis* males invariably divide into 1 functional spermatid and 1 DNA-containing RB as do the majority (∼80%, *N* = 79) of those produced by *mas-1* pseudomales (Figs. 1b and 3a).

To better understand these anaphase patterns, we focused on spermatocytes which were oriented as if we were looking down the barrel of the meiotic spindle to visualize the number of Hoechst-stained bodies (chromosomes or chromatids) on the metaphase plate (Fig. 3c). Previous studies of wild-type *A. rhodensis* found that males have 6 pairs of autosomes and 1 X chromosome (Shakes *et al.* 2011; Tandonnet *et al.* 2018). However, in the spermatocytes of XX pseudomales, we observed some metaphase plates only with autosomes (0X) as well as metaphase plates with 1–4X chromatids. Finding metaphase plates with extra X chromosomes/chromatids corroborated our finding of anaphase II figures with 3 or more X chromosomes. Finding metaphase plates with 6 autosomes and no Xs explains the ability of *mas-1* pseudomales to produce 0X spermatids.

Together with our previous studies of wild-type segregation patterns (Tandonnet *et al.* 2018), these cytological and brood composition studies of *mas-1* pseudomales suggest potential models of the observed patterns and how sperm with variable numbers of X chromosomes might be produced (Fig. 3e) If the 2 X chromosomes pair and recombine, they should undergo normal Mendelian segregation and produce 1X sperm. We did identify partitioning secondary spermatocytes with the form of a “classic” budding figure and seemingly equal anaphase chromatin masses; however, in the absence of live imaging, we could not determine whether such figures produce 2 viable sperm or ultimately collapse to form a single function sperm (Fig. 3e). In a second scenario, we predicted the 2 X chromosomes would behave like those previously described for wild-type *A. rhodensis* hermaphrodites; the X chromosomes would not pair and recombine during meiotic prophase. During meiosis I, they would separate into sister chromatids; and during meiosis II, the 2 nonsister X chromatids would segregate to 1 functional sperm. Alternatively, these X chromatids might segregate symmetrically to the sister spermatids. A third pattern would mimic that of X chromosomes during oogenesis in wild-type *A. rhodensis* hermaphrodites; all the X chromosomes would segregate to 1 second spermatocyte (or the first meiotic polar body of an oocyte) during meiosis I. This scenario would be a potential source of 0X sperm as well as anaphase II figures with multiple variations on segregating >2X chromatids. Lastly, *mas-1* spermatocytes could plausibly segregate unequal numbers of X chromatids to the 2 secondary spermatocytes during anaphase I. This scenario could generate both the observed secondary spermatocytes with a single X chromatid and many additional patterns.

### Postmeiotic sperm components cosegregate with the X chromosomes

To test our hypothesis that X chromatids were specifying the orientation of organelle segregation, we used immunocytology to examine the pattern of segregation of cytoplasmic components relative to the X-chromosome segregation in fixed sperm spreads of wild-type and *mas-1* males. We chose cytoplasmic components that are essential for postmeiotic sperm (MSP and mitochondria) and others that are discarded into residual bodies (endoplasmic reticulum and α-tubulin). The MSP drives nematode sperm motility; but within spermatocytes, MSP is contained within organelles called fibrous bodies (FBs) (Smith 2014). Except for mitochondria, the distribution of MSP, α-tubulin and endoplasmic reticulum (ER) in wild-type XO *A. rhodensis* spermatocytes has been previously described (Shakes *et al.* 2011; Winter *et al.* 2017).

During the first meiotic division of both wild-type XO and XX *mas-1* spermatocytes; organelles partitioned equally to both secondary spermatocytes (Supplementary Fig. 2, s). Notably, organelles segregate symmetrically during the first meiotic division of *mas-1* spermatocytes, even when the chromosomes are segregated asymmetrically (Supplementary Fig. 2, a).

**Fig. 4.**
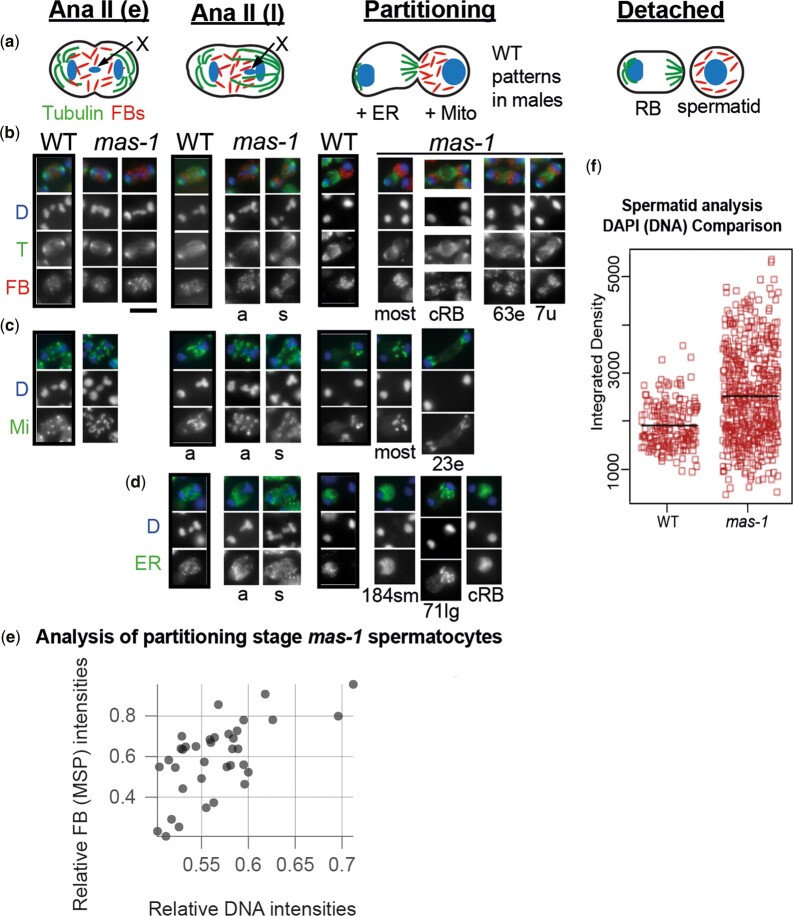
X-chromatid segregation patterns largely predict the pattern of microtubule and organelle partitioning in *mas-1* spermatocytes. a) Schematic of stage-specific spermatocytes in wild-type *A. rhodensis* males highlighting the chromosomes (blue), microtubules (green), and FBs (red) adapted from Winter *et al.* (2017). During early anaphase II (Ana II e), the X chromatid is centered, and the spindle is symmetric. During late anaphase II (Ana II l), the X chromosome segregates to 1 side, and microtubules elongate on that side. During partitioning, the FBs and mitochondria partition to the X-bearing side. The centrosome on the X-side deactivates. ER partitions to the non-X side (residual body side) which retains its original centrosome as noncentrosomal microtubules emanate from the RB-spermatid boundary. Ultimately, the spermatid detaches from the residual body. Staged spermatocytes from sperm spreads of XO wild-type males (boxed) and XX *mas-1* pseudomales stained with DAPI (d) and labeled with antibodies against (b) α-tubulin (T) and the FB marker MSP, (c) the mitochondrial (Mi) β-subunit of ATP synthase, and (d) the ER-specific antibody CYP33E1 (cytochrome P450 family). Spermatocytes are indicated as having X chromatids that segregate either asymmetrically(a) or symmetrically(s). Images (b)–(d) enlarged 1.5× for visibility. Abbreviations: even (e), uneven (u), small chromatin mass (sm), and large chromatin mass (lg). Numbers under images indicate the number of examples found. Scale bar= 5 µm*.* e) Scatter plot of a subset of partitioning stage *mas-1* spermatocytes in which FBs are partitioning to both sides. Integrated intensities of larger DAPI-stained chromatin mass/sum of the 2 masses plotted relative to the distribution MSP-labeled FBs. 0.5 indicates equal partitioning and 1 indicates partitioning to 1 side. Pearson correlation coefficient = 0.63. f) Quantification of DAPI in MSP-containing spermatids in wild-type and *mas-1* males.

In wild-type XO males, secondary spermatocytes undergo a stereotypic series of cytological events which we document again here (Winter *et al.* 2017) (Fig. 4, a and b). During early anaphase II, the autosomes segregate to opposite poles, the microtubule spindle remains symmetric, the X chromatid is central, and the organelles are centrally distributed. During late anaphase II, as the X moves to 1 side, the microtubules elongate specifically on that side. Once the X incorporates into the autosomal chromatin mass, organelles needed by the sperm like the MSP-containing FBs partition to the X-bearing side. Then, the centrosome on the X-bearing side deactivates while its microtubules and gamma–tubulin complexes move to the boundary between the sperm and the forming RB. Mitochondria (Fig. 4c) follow a similar pattern to that of the FBs. In contrast, the endoplasmic reticulum (Fig. 4d) partitions both later in the process and ultimately to the RB.

In *mas-1* XX pseudomales, organelle partitioning within secondary spermatocytes exhibited a more variable pattern (Fig. 4, b–d). During early anaphase II, the spindles were symmetric as was the distribution of FBs (Fig. 4b) and mitochondria (Fig. 4c). However, the central X chromosomes were either stacked on top of each other as had been previously reported in the spermatocytes of wild-type hermaphrodites or spread out centrally in a novel pattern (Fig. 4b). During late anaphase, the organelles remained centrally distributed, regardless of whether the X chromatids were segregating asymmetrically (a) to 1 side or symmetrically (s) to both sides (Fig. 4, b–d). However, the microtubules mirrored the X chromosomes patterns; they became asymmetric when the X chromatids segregated asymmetrically and remained more symmetric when the X chromatids segregated symmetrically.

During the partitioning stage, we could score chromatin masses as symmetric or asymmetric. However, without the ability to specifically mark the X chromosome, we could not specifically assess how many X chromatids were segregated to each side in *mas-1* spermatocytes. Most partitioning stage spermatocytes had asymmetric chromatin masses. In these cases, the FBs and mitochondria partitioned completely or mostly to the larger mass (Fig. 4, b and c). To further explore this relationship, we examined the subset of partitioning stage spermatocytes in which FBs partitioned to both sides and in which both DNA masses and the FBs were in clear focus (*N* = 36). For these we examined the correlation between asymmetry of the DNA masses (integrated intensity of the larger DAPI-stained chromatin mass/sum of the 2 chromatin masses) and a corresponding measurement of the MSP-labeled FBs on each side (Fig. 4e). Overall, there was a positive (*r *=* *0.63; *N* = 36) between the measured asymmetries in DNA and FB (MSP) intensities. FBs generally (27/36) but not always (3/36) exhibited biased partitioning to the side with more DNA. Notably, when the DNA masses were symmetric (close to 0.5) reflecting either symmetric X-chromosome segregation or the lack of Xs from a prior anaphase I loss (6/36), FB partitioning appeared both unequal and seemingly random. In contrast to the mitochondria and FB patterns, the ER marker exhibited a variable pattern of either segregating to the smaller chromatin mass as expected or, in an unexpected pattern, to the larger chromatin mass (Fig. 4d). Also, as anticipated, some partitioning stage *mas-1* spermatocytes formed bipolar figures with cRBs. In these cells, FBs and mitochondria partitioned symmetrically toward both chromatin masses (Fig. 4, b and c), ER components segregated to the cRB (Fig. 4d), and microtubule foci formed at both sperm-RB boundaries (Fig. 4b). In other cases, when organelles partitioned to both sides (Fig. 4b), the microtubule foci were distinct from either the 1 shifted foci pattern (most) or 2 shifted foci pattern (cRB).

To determine which cellular products of the partitioning stage formed spermatids rather than degrading as residual bodies, we analyzed the DNA content of spermatids that had fully detached from residual bodies and possessed both compacted DNA and MSP. In those cells, the range of DNA intensity and size of those sperm was broader than in wild-type XO males (Fig. 4f), confirming that *mas-1* males make sperm with both lower and higher DNA content than wild-type males, differences which presumably reflect variable numbers of X chromatids.

### In wild-type XX hermaphrodites, rare 1X sperm originate from specific spermatogonial clusters and distinctive patterns of X chromosome and organelle partitioning

**Fig. 5.**
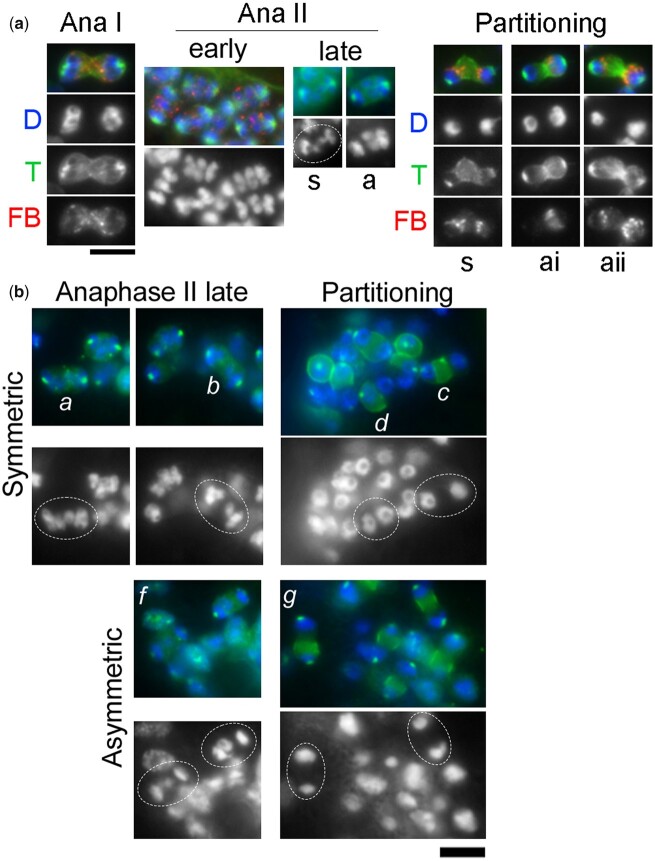
Two patterns of spermatocyte divisions in wild-type hermaphrodites. A) Staged spermatocytes from wild-type XX *A. rhodensis* hermaphrodites stained with DAPI (D) and labeled with antibodies against α-tubulin (T) and the FB marker MSP (M) Spermatocytes are indicated as having X chromatids that segregate either symmetrically (s) or asymmetrically (a). In partitioning spermatocytes with asymmetric DNA masses, either all (i) or the majority (ii) of the FBs partitioned to the larger chromatin mass. B) Spermatocytes clusters labeled with anti-sumo antibody, anaphase II spermatocytes. Scale bar = 5 µm. In symmetric clusters (a, b) the X chromatids segregate to both poles in equal-sized chromatin masses (c, d) during the partitioning stage. In asymmetric clusters (f, g), both X chromatids segregate to 1 pole and the partitioning stage chromatin masses are uneven.

Self-fertilizing wild-type *A. rhodensis* hermaphrodites produce XO male offspring in their early brood (Chaudhuri *et al.* 2015; Tandonnet *et al.* 2018; Supplementary Fig. 1), but the cytological underpinnings of how rare 1X hermaphrodite sperm form has not been previously documented. We hypothesized that the formation of rare 1X sperm would be associated with distinct spermatogonial clusters that enable *A. rhodensis* hermaphrodites to produce sperm throughout adulthood (McCaig *et al.* 2017). This mode of gonad organization means that the same stage, same spermatogonial cluster-derived spermatocytes would be physically clustered together unless separated during the process of gonad dissection (Fig. 5A—early anaphase II). By analyzing hermaphrodites that were either young adults or in the preceding molting stage, we maximized our chance of capturing at least some anaphase II or partitioning stage spermatocytes that were fated to generate 1X rather than 2X sperm. In fixed samples colabeled with DAPI, antitubulin, and anti-MSP, anaphase I and early anaphase II patterns were as previously reported (Fig. 5A). Chromosome segregation during anaphase I was symmetric and lagging chromosomes were not observed. During early anaphase II, 2 X chromatids typically lagged in the center and appeared to be stacked on top of each other, suggesting they were independently attached to the spindle. We also observed rare patterns of chromosome segregation in late anaphase II in which the Xs segregated symmetrically (s) to the 2 poles rather than the typical pattern of segregating asymmetrically (a) to 1 pole. During the partitioning stage, rare spermatocytes with symmetric (s) chromosome segregation exhibited bipolar budding figures and organelles partitioning toward both poles; although within the same spermatogonial cluster, we often observed cells that appeared to have resolved into a unipolar pattern as proposed in Fig. 3d. In spermatocytes with the asymmetric (a) pattern of both X chromatids segregating to 1 pole, organelles segregated completely (ai) or mostly (aii) to the 2X side. Parallel studies using anti-sumo antibodies to label chromosome and actin-patterns (Fig. 5B), confirmed these results. Spermatocytes from symmetric clusters (a, b) have X chromatids segregating to both poles and equal-sized chromatin masses (c, d) during the partitioning stage, whereas in asymmetric clusters (f, g), both X chromatids segregate to 1 pole and the partitioning stage chromatin masses are uneven. These results not only reveal how variations in anaphase II X-chromosome segregation patterns enable XX hermaphrodites to produce either 1X or 2X sperm, but they also show that organelle partitioning orients toward the late segregating X chromosomes.

## Discussion

The initial observation of a few male offspring derived from *Auanema* male and female crosses revealed male spermatocytes undergo a modified spermatogenesis resulting in functional X-bearing sperm and residual bodies with the non-X genetic complement (Shakes *et al.* 2011; Winter *et al.* 2017). This meiotic variation generates biased sex ratios, which provides adaptive advantages in certain ecological circumstances (Van Goor, Herre, *et al.* 2022). In the present study, we examined X-chromosome behavior and organelle partitioning in 2 exceptional conditions: XX *mas-1* pseudomales and young wild-type hermaphrodites which can make both 1X and 2X sperm. Our study resulted in 3 key findings: (1) in the abnormal context of XX male spermatogenesis which has not been subjected to evolutionarily adaptation, patterns of X-chromosome segregation were even more variable, (2) during wild-type hermaphrodite spermatogenesis, 1X and 2X sperm are produced in distinct spermatogonial clusters and arise from 2 different patterns of X-chromatid segregation during anaphase II, and (3) even with the additional variations, the patterns of lagging X-chromatid segregation during anaphase II largely predicted the orientation of organelle partitioning. However, when X chromatids segregated symmetrically or were potentially absent, FBs often partitioned to both sides in less predictable patterns. Both these findings are consistent with our hypothesis that X chromatids serve a directive role in orienting the direction of organelle partitioning.

Identification and isolation of the sex determination mutant *mas-1* allowed us to study patterns of X-chromosome segregation in the nonadapted context of an XX male. Since the pseudomales were fertile, analysis of the sired offspring enabled us to assess the composition of the functional/competitive sperm as ¾ 1X, ¼ 0X, and a small number of 2X sperm (Fig. 2f). However, our cytological analysis of both spermatocytes and mature spermatids from isolated pseudomale gonads indicated a much higher fraction of 2–4X sperm were being produced. This result suggested that many of these sperm were either nonfunctional or noncompetitive and thus failed to contribute to the production of either viable offspring or dead embryos. Some of this discrepancy can be explained by the likely overrepresentation in fixed sperm spreads of late anaphase figures with multiple X chromatids, since the presence of extra and odd numbers of chromatids presumably prolongs anaphase II. Conversely, secondary spermatocytes without X chromatids were underrepresented as the absence of lagging X presumably shortens anaphase II.

Prior to this study, we knew that, in *A. rhodensis*, spermatocyte meiosis yielded 2 rather than 4 functional sperm and that organelles required for sperm function partitioned to X-bearing chromatin side; the side with the single X chromatid in male spermatocytes or the 2 nonsister X chromatids in hermaphrodite spermatocytes (Shakes *et al.* 2011; Tandonnet *et al.* 2018). The partitioning process itself is a universal part of RB formation, and its relationship to the anaphase II axis is a feature of nematode spermatogenesis. However, the conversion of a bipolar to a unipolar process is unique either to *Auanema* or potentially to nematode species with highly diminutive spermatocytes. This study addressed whether, as we studied additional variations in anaphase II X-chromatid segregation patterns, would we continue to see a correlation between the pattern of lagging X chromatids and the orientation of organelle partitioning. With the important limitations that we lacked the tools to either directly mark the X chromosome or live image organelle partitioning through time, essential organelles partitioned toward the X chromatids in both the spermatocytes of XX pseudomales and in early spermatocytes of wild-type hermaphrodites. When all the Xs segregated to 1 side, FBs and mitochondria typically did as well. When Xs segregated symmetrically or may not have been present at all, FBs and mitochondria are often partitioned to both sides but unequally and unpredictably. Additional technical limitations of our studies included using DNA fluorescence as a proxy for DNA amounts when differential DNA packing could impact DAPI binding and performing the analysis of single-focal plane images, which included most but perhaps not all of the full DNA mass and likely not all of the FBs. Despite these technical limitations, these studies are the first to document bipolar budding figures in *A. rhodensis*, which were observed to accompany symmetric X-chromatid segregation in both *mas-1* spermatocytes and early wild-type hermaphrodite spermatocytes. Still unknown is whether the observed bipolar budding figures ever yield 2 functional sperm or whether they must secondarily restructure to form a single functional sperm. Presumably, sperm require a minimal number of mitochondria to power the sperm and enough MSP and cytoplasm to form a pseudopod that can support motility and traction within the female reproductive tract.

A notable exception to our finding that proportional DNA mass correlates with the direction of organelle partitioning was that in 27% (*N* = 255) of partitioning stage *mas-1* spermatocytes, ER markers partitioned “incorrectly” toward the larger chromatin mass, a situation which may compromise normal sperm function. One potential explanation is that ER partitioning is known to occur late in the partitioning process and correlates with microtubules moving to the RB (Winter *et al.* 2017). Although we did not directly study the relationship between ER and microtubule patterns, we did document unusual microtubule patterns in partitioning stage *mas-1* spermatocytes that differ from either the unipolar or bipolar reorganization of a microtubule organizing center to the spermatid-RB boundary (Fig. 4b). Although *mas-1* pseudomales are fertile, defects in organelle partitioning may contribute to a subset of their sperm being fertilization incompetent. If inappropriately partitioned ER is a proxy for the RB material, inappropriate amounts of RB material could be both detrimental and/or trigger apoptosis (Huang *et al.* 2012).

The finding that most *mas-1* offspring are the product of 1X and 0X sperm combined with the observed cytology suggests that spermatocytes in *mas-1* XX males possess the full range of *A. rhodensis* genetic “tricks.” As occurs in XX hermaphrodite oocyte meiosis (Tandonnet *et al.* 2018), the 2 Xs can segregate into one of the 2 secondary spermatocytes during anaphase I, and we suspect that the resulting secondary spermatocytes without Xs are the most likely source of 0X sperm. Conversely, 1X sperm that accounts for most of the progeny sired by *mas-1* pseudomales could potentially arise from either standard Mendelian patterns as occurs in XX female oocyte meiosis or from the symmetric segregation of nonsister X chromatids during anaphase II that we documented for the first time in rare, early spermatocytes of wild-type hermaphrodites. In a novel pattern, spermatocytes of *mas-1* pseudomales exhibit diverse patterns of X-chromatid segregation during anaphase II, reflecting both differing numbers of X chromatids and differential associations (e.g. stacked X chromatids vs singlets distributed along the spindle axis). These anaphase II variations presumably reflect stochastic variations in meiotic spindle dynamics and seem nonadapted, as the numerous sperm with 2 or more X observed cytologically are not represented in the broods sired by *mas-1* males. In future studies, it will be interesting to determine exactly how these X chromatids are attached to the meiotic spindle, given the novel features of lagging X-chromosome segregation mechanisms recently described in *C. elegans* males (Fabig *et al.* 2020). In addition, now that we have determined that 1X sperm in wild-type *A. rhodensis* hermaphrodites are generated within specific spermatogonial clusters, it will be interesting to explore whether the numbers of these rare clusters increase adaptively in response to specific environmental conditions.

This study represents an important next step in the analysis of this fascinating genetic system in which noncanonical patterns of X-chromatid segregation support sex ratios that are highly biased against males. The coupling of postmeiotic organelle partitioning to the pattern of late segregating X chromatids in *A. rhodensis* then allows for the efficient conversion of a normally bipolar partitioning process into a unipolar process. At the same time, the establishment of this linkage appears to have compromised the efficiency of organelle partitioning in the context of either the *mas-1*-specific incidences of having no X chromatids or during symmetric X-chromosome segregation, which in wild-type animals is restricted to a rare subset of hermaphrodite spermatogonial clusters.

## Supplementary Material

iyac159_Supplementary_Data

## Data Availability

Strains are available upon request. The authors affirm that all data necessary for confirming the conclusions of the article are present within the article, figures, and tables. Supplemental material is available at GENETICS online.
