# Supplementary material for: Lagging X chromatids specify the orientation of asymmetric organelle partitioning in XX spermatocytes of Auanema rhodensis
Source: Genetics. 2022 Oct 18;222(4):iyac159. doi: 10.1093/genetics/iyac159 (PMC9713428; doi:10.1093/genetics/iyac159)
Supplement: iyac159_Supplementary_Data [file iyac159_supplementary_data.docx]

**Supplemental Data**

**Lagging X chromatids specify the orientation of asymmetric organelle partitioning in XX spermatocytes of *Auanema rhodensis***

Talal Al-Yazeedi^1,3^, Emily L. Xu^2,3^, Jasmin Kaur^1^, Diane Shakes^2#^, and Andre Pires-daSilva^1#^

^1^School of Life Sciences, University of Warwick, Coventry, CV4 7AL, UK

^2^Department of Biology, William & Mary, Williamsburg, VA 23187, USA

^3^Co-first authors

^#^Co-corresponding authors

**Figure S1**. **Maintenance of *mas-1* strain**. To distinguish between wild-type and heterozygous hermaphrodites and to maximize likelihood of choosing *mas-1/mas-1* males for experiments, we separated broods into early brood, mid-brood, and late brood by transferring the heterozygous parent every 24 hours. Hermaphrodite (parent dauer), and hermaphrodites/females (offspring) in light gray. Wild type and *mas-1* pseudomales in dark blue. Wild-type males are produced early in the brood while homozygous *mas-1* males are produced throughout the brood.

**
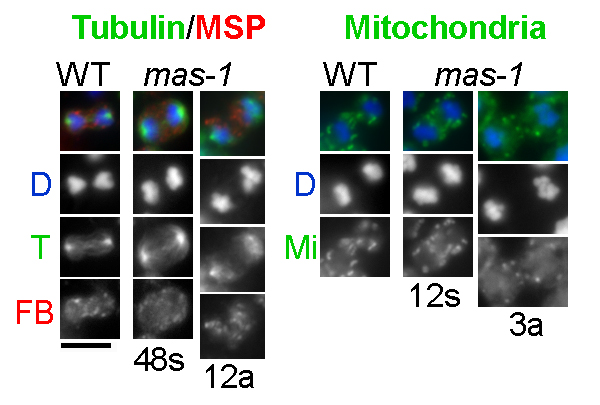
**

**Figure S2. Cytoplasmic components partition evenly during anaphase I.** Anaphase I spermatocytes from sperm spreads of XO wild type (WT) males and XX *mas-1* pseudomales stained with DAPI (blue) and antibodies against ⍺-tubulin (green) and the FB marker MSP (red) or the mitochondrial beta-subunit of ATP synthase (green). Scale bar = 5 µm. Numbers of observed spermatocytes with symmetric (s) or asymmetric (a) segregation of their X chromosomes are indicated below each image.
